# Identification of the cuproptosis-related molecular subtypes and an immunotherapy prognostic model in hepatocellular carcinoma

**DOI:** 10.1186/s12859-022-04997-0

**Published:** 2022-11-16

**Authors:** Li Zhang, Jingwei Xu, Xiufeng Chu, Hongqiao Zhang, Xueyuan Yao, Jian Zhang, Yanwei Guo

**Affiliations:** Department of Oncology, The Fifth Affiliated Hospital of Zhengzhou University, Zhengzhou, China

**Keywords:** Hepatocellular carcinoma (HCC), Cuproptosis, Subtype, Prognostic model, Bioinformatics, Machine learning

## Abstract

**Background:**

Cuproptosis, a newly discovered mode of cell death, has been less studied in hepatocellular carcinoma (HCC). Exploring the molecular characteristics of different subtypes of HCC based on cuproptosis-related genes (CRGs) is meaningful to HCC. In addition, immunotherapy plays a pivotal role in treating HCC. Exploring the sensitivity of immunotherapy and building predictive models are critical for HCC.

**Methods:**

The 357 HCC samples from the TCGA database were classified into three subtypes, Cluster 1, Cluster 2, and Cluster 3, based on the expression levels of ten CRGs genes using consensus clustering. Six machine learning algorithms were used to build models that identified the three subtypes. The molecular features of the three subtypes were analyzed and compared from some perspectives. Moreover, based on the differentially expressed genes (DEGs) between Cluster 1 and Cluster 3, a prognostic scoring model was constructed using LASSO regression and Cox regression, and the scoring model was used to predict the efficacy of immunotherapy in the IMvigor210 cohort.

**Results:**

Cluster 3 had the worst overall survival compared to Cluster 1 and Cluster 2 (*P* = 0.0048). The AUC of the Catboost model used to identify Cluster 3 was 0.959. Cluster 3 was significantly different from the other two subtypes in gene mutation, tumor mutation burden, tumor microenvironment, the expression of immune checkpoint inhibitor genes and *N*^6^-methyladenosine regulatory genes, and the sensitivity to sorafenib. We believe Cluster 3 is more sensitive to immunotherapy from the above analysis results. Therefore, based on the DEGs between Cluster 1 and Cluster 3, we obtained a 7-gene scoring prognostic model, which achieved meaningful results in predicting immunotherapy efficacy in the IMvigor210 cohort (*P* = 0.013).

**Conclusions:**

Our study provides new ideas for molecular characterization and immunotherapy of HCC from machine learning and bioinformatics. Moreover, we successfully constructed a prognostic model of immunotherapy.

**Supplementary Information:**

The online version contains supplementary material available at 10.1186/s12859-022-04997-0.

## Background

Hepatocellular carcinoma (HCC), one of the deadliest cancers, remains a global health challenge, and its incidence has been growing worldwide in recent years [1]. Many treatments in HCC include surgery, ablation, transarterial chemoembolization, transarterial radioembolization, and systemic therapy. Furthermore, since the advent of sorafenib, research on molecularly targeted therapy and immunotherapy has deepened, significantly improving patients' overall and progression-free survival, and has shown promise in HCC [2]. Despite this, the prognosis for HCC remains poor. Therefore, identifying novel molecular subtypes to develop individualized drug regimens and predict the effect of immunotherapy is of great significance for the prognosis of HCC.

Several previous studies have revealed the relationship between copper and cancer. Some scholars have found that the combined application of disulfiram and copper has an anti-tumor effect [3–5]. A study demonstrates the relationship between copper-dependent cell proliferation and various cellular processes [6]. Another study revealed that intra-tumoral copper levels influence *PD-L1* expression in cancer cells, and copper regulates critical signaling pathways mediating *PD-L1*-driven cancer immune evasion [7]. Recently, a major study showed that copper-dependent death occurs by directly binding copper to lipoylated components of the tricarboxylic acid cycle [8]. The combination leads to lipoylated protein aggregation and subsequent iron-sulfur cluster protein loss, leading to cell death [8]. This study proposes to name this new form of death cuproptosis.

Cuproptosis provides a new direction for treating HCC, but few related studies exist between cuproptosis and HCC. A recent study demonstrated that the cuproptosis-related gene *FDX1* plays an essential role in the prognosis of HCC and successfully developed a cuproptosis-related risk score that can predict the efficiency of sorafenib and the non-responsiveness to transcatheter arterial chemoembolization in HCC patients [9]. Using machine learning methods to classify HCC has become crucial for studying HCC. It can explore different molecular characteristics of HCC, provide new therapeutic targets for HCC, and guide individualized treatment plans for HCC patients [10–12]. At the same time, since immunotherapy is becoming more and more critical in HCC, it is meaningful to predict the efficacy of immunotherapy based on the signature score of cuproptosis. A flowchart of this study is provided in Additional file 1: Fig. S1.

## Methods

### Datasets

The mRNA expression data and clinical information were obtained from UCSC Xena^1^ (GDC TCGA Liver Cancer) [13]. We got 357 HCC samples by excluding data with no survival information and the survival time of fewer than 30 days. Expression and clinical data of IMvigor210 [14] were used to validate the immunotherapy efficacy of the cuproptosis signature score, and 298 samples were obtained.

### Identification of molecular subtypes

Ten genes were identified with important significance in cuproptosis, among which *MTF1*, *GLS*, and *CDKN2A* have negative regulatory effects, and *FDX1*, *DLD*, *LIPT1*, *LIAS*, *DLAT*, *PDHA1*, and *PDHB* have positive regulatory effects [8].

Unsupervised clustering analysis operated by consensus clustering is a reliable technique to identify groups of unknown but common biological characteristics in cancer research. The R package “ConsensusClusterPlus” was used for this analysis to classify patients into distinct molecular subtypes according to the expression matrix of cuproptosis-related genes (CRGs). Some parameters are as follows: maxK = 10, reps = 100, clusterAlg = “pam”, and distance = “pearson”. The stability of the curve in the empirical cumulative distribution function (CDF) plot and the degree of cleanliness in the consensus matrix (CM) plot determine the number of groups (k) for clustering [15].

Survival curves plotted by the “survival” R package were employed to assess clinical significance between subtypes. The “Rtsne” R package and the “UMAP” R package were used to operate and visualize the t-distributed stochastic neighbor embedding (t-SNE) and the uniform manifold approximation and projection (UMAP) based on the consensus clustering results, respectively. The distribution maps of these two-dimensionality reduction algorithms were used to observe the confounding of subtype samples obtained from consensus clustering.

### Construction of predicting model

According to the results of consensus clustering, six machine learning methods were used for supervised machine learning, including XGBoost, CatBoost, support vector machine (SVM), random forest (RF), logistic regression (LR), and neural network (NNET). We utilized the “xgboost” R package, the “catboost” R package, and the “mlr3” R package to execute these algorithms. By comparing the ROC curves, we choose the most appropriate algorithm to build the model to predict subtypes based on CRGs.

### Analysis of somatic variants

We downloaded MuTect2 Variant Aggregation and Masking files from UCSC Xena. The “MAftools” R package [16] was used to explore different subtypes’ gene mutations and enrichment of oncogene pathways. We also calculated the value of tumor mutation burden (TMB) in different subtypes, a valuable biomarker that can predict the efficacy of immunotherapy [17].

### Analysis of tumor microenvironment

Tumor microenvironment (TME) is closely related to immune escape in solid tumors. Therefore, analyzing the infiltration of immune cells in different HCC subtypes is of great significance for predicting the efficacy of immunotherapy. This part used four algorithms, including TIMER, CIBERSORT, quanTIseq, and MCP-counter, to evaluate the immune cell infiltration score [18, 19].

### Expression of immune checkpoint inhibitor genes and N^6^-methyladenosine regulatory genes

The expression of immune checkpoint inhibitor genes correlates with the efficacy of immunotherapy. The change in the *N*^6^-Methyladenosine (m6A) level is involved in the occurrence and development of tumors. We explored the expression differences of these genes between different subtypes. We obtained six immune checkpoint inhibitor genes and 15 m6A regulatory genes from previous studies [20, 21] and the (GeneCards database^2^). These genes are provided in Additional file 3: Table S1.

### Prediction of drug sensitivity

Sorafenib, a landmark tyrosine kinase inhibitor, was the first systemic therapy to show a survival benefit in advanced HCC [22]. The “pRRophetic” R package was used to predict the drug sensitivity of sorafenib in different subtypes [23]. This R package makes predictions based on the 50% inhibitory concentration (IC50).

### Exploration of molecular mechanisms

We performed Gene Ontology (GO) enrichment analysis and Kyoto Encyclopedia (KEGG) pathway analysis in different subtypes using the gene set variation analysis (GSVA) method based on the “GSVA” R package [24]. GO, and KEGG gene sets were obtained from the molecular signature database (MSigDB^3^). The GSVA scores for each subtype are presented as a heatmap by the “pheatmap” R package.

### Establishment of the cuproptosis-based prognostic signature score

Based on the survival analysis, TMB, and TME, we selected two subtypes with both significant survival differences and possible differences in immunotherapy efficacy to analyze differentially expressed genes (DEGs) utilizing the “DESeq2” R package [25] (|log2FoldChange|> 1, adjusted *P*-value < 0.05). The overfitting between the DEGs was removed by the least absolute shrinkage and selection operator (LASSO) algorithm to reduce the scope of DEGs. Multivariate Cox regression was used on the results of the LASSO regression, and prognostic genes with *P*-values less than 0.05 were included in the model. Cox and LASSO regression are implemented based on the “glmnet” R package [26]. The formula for signature score is as follows:

Signature score = the expression of each gene *_e_ (the coefficient of multivariate Cox regression).

Calibration curves [27], time-dependent ROC curves [28], and decision curve analysis (DCA) [29] were employed to assess the effect of the signature score model.

Next, the signature score of each sample in IMvigor210 was calculated. Grouped according to the median of the signature score and compared the efficacy of immunotherapy between the two groups.

### Statistical analysis

All statistical analyses were performed by R version 4.0.5 and its appropriate packages. The Shapiro test was used to check whether the data is normally distributed. Survival curves were drawn using the Kaplan–Meier method and compared by the log-rank test. *P*—values < 0.05 were statistically significant.

## Results

### Identification and validation of molecular subtypes

**Fig. 1:**
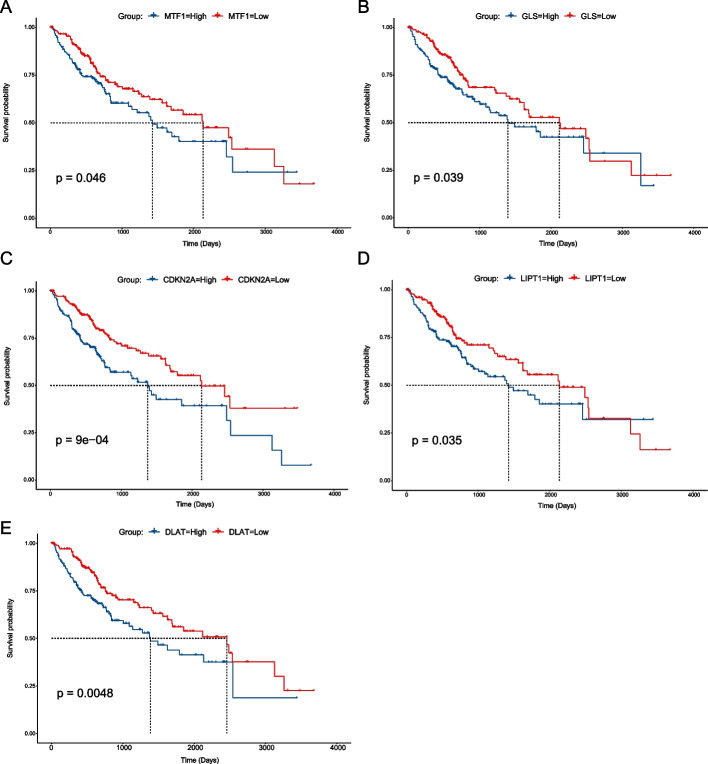
Survival analysis of the ten cuproptosis-related genes. **A**–**E** Respective survival curves of *MTF1*, *GLS*, *CDKN2A*, *LIPT1*, and *DLAT* in HCC samples (group by the median of gene expression). The dotted line represents the median survival time of the different groups

First, we explored the survival significance and relevance of the ten CRGs in the HCC samples. Grouped according to these ten genes' median gene expression levels, we performed survival analysis on these high and low expression groups. The results showed that low expression of *MTF1* (*P* = 0.046), *GLS* (*P* = 0.039), *CDKN2A* (*P* < 0.0001), *LIPT1* (*P* = 0.035), and *DLAT* (*P* = 0.0048) improve the overall survival (Fig. 1A–E). To explore the relationship of these ten genes, we performed protein–protein interactions (PPI) analysis and expression correlation analysis on them. Additional file 2: Fig. S2A shows that *LIAS* and *DLD* are core genes in the protein–protein interactions [30]. Additional file 2: Fig. S2B displays that the expression of these ten CRGs in HCC samples is almost all positively correlated, and the highest correlation between genes expressions is 0.56.

**Fig. 2:**
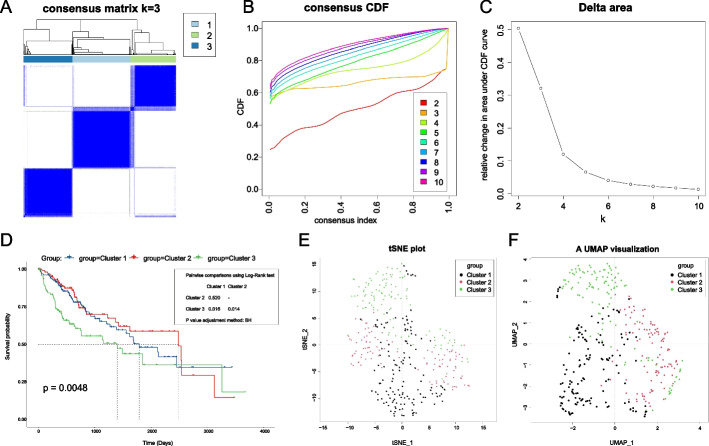
The identification and verification of cuproptosis-related HCC subtypes in HCCcohort. **A** Three cuproptosis-related molecular subtypes were recognized via unsupervised consensus clustering. **B** Empirical cumulative distribution function (CDF) plots displayed the consensus distributions for each k. **C** The delta area score showed relative growth in cluster stability. **D** Survival curves of the three subtypes. **E**, **F** Visualization of the analysis of t-distributed stochastic neighbor embedding (t-SNE) and uniform manifold approximation and projection (UMAP), respectively

**Fig. 3:**
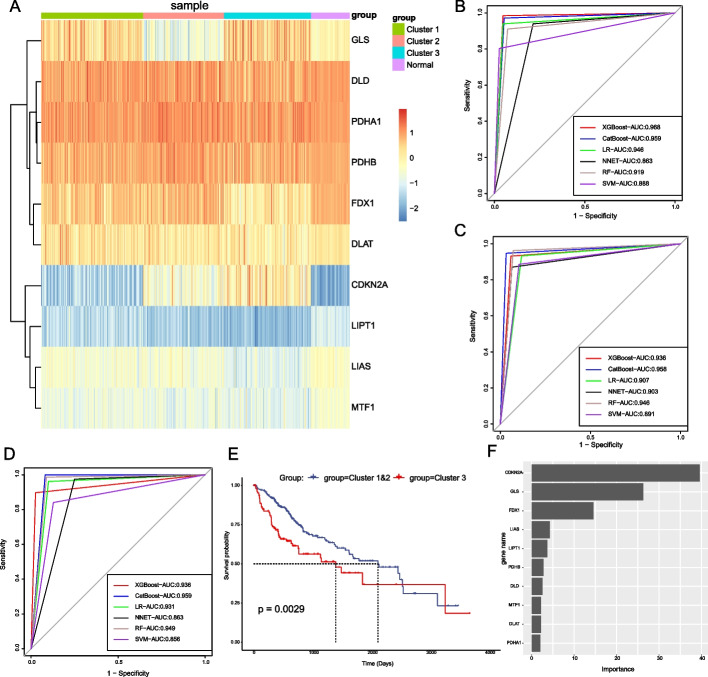
Establishment of six machine learning models for the three subtypes. **A** The heatmap of expression of the ten cuproptosis-related genes between different subtypes and normal tissues. **B–D** ROC curves of confusion matrices for Cluster 1, Cluster 2, and Cluster 3. **E** Survival curves derived from Cluster 3 were identified in 357 HCC samples using the catboost model. **F** Ranking of gene importance affecting the catboost model identifying Cluster 3

We next performed consensus clustering of 357 HCC samples. We found that K = 3 is the optimal number of clustering groups in increasing the clustering variable (K) from 2 to 10. When K = 3, the inter-group correlation is the lowest, the intra-group correlation is the highest (Fig. 2A), and the CDF curve is the flattest (Fig. 2B). The change in K from 3 to 4 is the part with the most considerable change in the area under the CDF curve (Fig. 2C). Therefore, K = 3, and choosing the number of groups to be three is the most appropriate. We named these three groups Cluster 1, Cluster 2, and Cluster 3. Survival analysis of these three groups indicated that the classification was clinically meaningful. There was no statistical significance in overall survival between Cluster 1 and Cluster 2, and Cluster 3 has the worst overall survival (Fig. 2D). UMAP and t-SNE revealed that Clusters 1 and 2 could not be clearly differentiated, and Cluster 3 is more clearly differentiated from the other two (Fig. 2E, F). Figure 3A shows the expression of the ten CRGs in the three subtypes and normal liver tissue. Additional file 4: Table S2 provides the clinical characteristics of these three subtypes.

### Construction of the machine learning predicting model

In this part, we built models that recognize these three subtypes based on supervised machine learning with six algorithms, including XGBoost, CatBoost, SVM, RF, LR, and NNET. When modeling each subtype, we used the “caret” R package to divide the 357 HCC samples into a 70% training set and a 30% validation set according to the principle of binary classification. We utilized the model from the training set to make predictions on the validation set and drew the ROC curve according to the actual and predicted results. After ten repetitions, the optimal algorithm model was judged according to the AUC value. We found that the XGBoost model was the best model for identifying Cluster 1 (Fig. 3B), and the CatBoost models were the best for identifying Cluster 2 and Cluster 3 (Fig. 3C, D). Since Cluster 3 has the worst overall survival rate, the clinical significance of distinguishing this subtype is more remarkable. We used the CatBoost model to determine Cluster 3 of 357 HCC samples and plotted the survival curve to verify the effect of the model (Fig. 3E). Figure 3F shows that *CDKN2A*, *GLS*, and *FDX1* are the most important genes identifying Cluster 3 in the CatBoost model calculated by the “catboost” R package.

### Analysis of the somatic variants

**Fig. 4:**
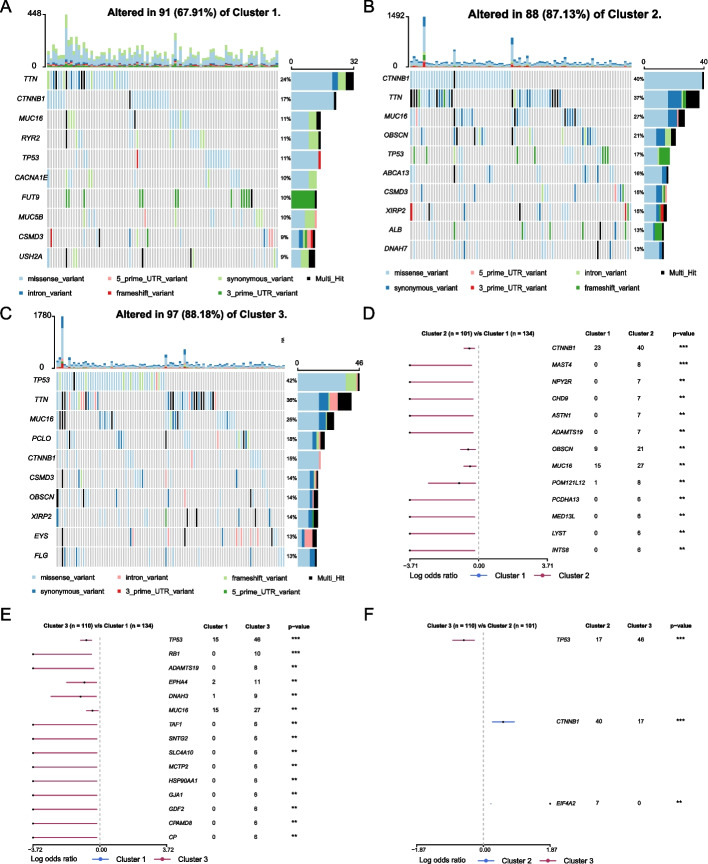
Mutation status analysis of the three subtypes. **A–C** Top 10 genes and mutation types by mutation ratio in Clusters 1, 2,and 3. **D**–**F** The forest plots of gene mutation of Cluster 1 vs. Cluster 2, Cluster 1 vs. Cluster 2, and Cluster 2 vs. Cluster 3, respectively

**Fig. 5:**
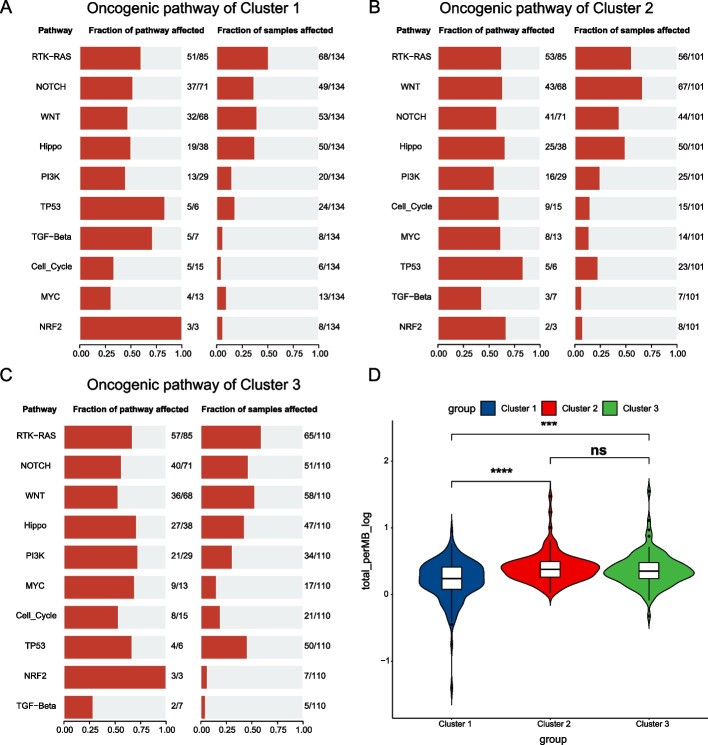
The analysis of oncogenic pathway and tumor mutation burden (TMB) in the three subtypes. **A**–**C** Oncogenic pathway enrichment analysis for Cluster 1, Cluster 2, and Cluster 3. **D** Comparison of TMB between the three subtypes. *P*-values were showed as: *P < 0.05: **P < 0.01; ***P < 0.001

Through the analysis of the mutated genes of these three subtypes, we found that the proportion of mutated genes in Cluster 1 is relatively small and mainly *TTN* (24%, Fig. 4A); Cluster 2 is dominated by *CTNNB1* mutation (40%, Fig. 4B); *TP53* mutation is the primary mutation in Cluster 3 (42%, Fig. 4C). We next compared the mutational profiles of these three subtypes with each other. Compared with Cluster 2 and Cluster 3, Cluster 1 had significantly fewer mutated genes (Fig. 4D, E), and there were fewer mutated differential genes between Cluster 2 and Cluster 3 (Fig. 4F), mainly *TP53* (*P* < 0.001) and *CTNNB1* (*P* < 0.001). Oncogene pathways enrichment analysis indicated that *RTK-RAS*, *NOTCH*, *WNT*, and *Hippo* were these three subtypes' main oncogenic signaling pathways (Fig. 5A–C). Compared with the other two subtypes, the pathway enrichment ratio of Cluster 1 was lower, and the percentage of the *TP53* path was distinctly higher in Cluster 3. We subsequently explored the TMB of the three subtypes. As shown in Fig. 5D, there were statistically significant differences in TMB between Cluster 1 and Cluster 2 (*P* < 0.0001), between Cluster 1 and Cluster 3 (*P* < 0.001), and not significant between Cluster 2 and Cluster 3. The above analysis shows that Cluster 1 is a mutation-less type, and Cluster 2 and Cluster 3 are mutation-rich types.

### Analysis of tumor microenvironment

**Fig. 6:**
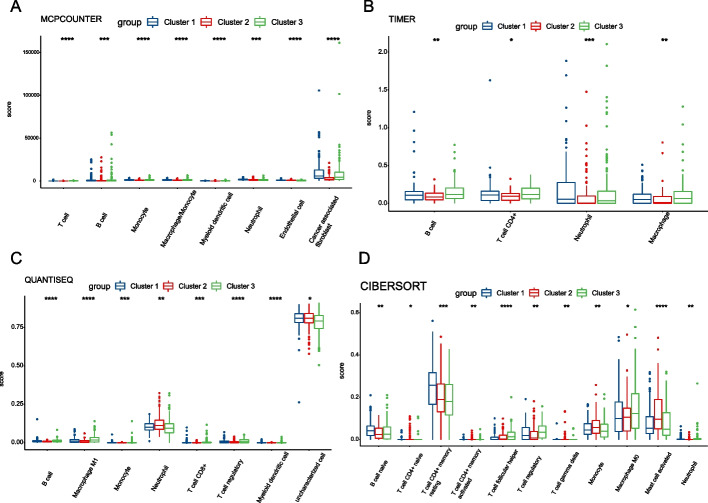
The analysis of tumor microenvironment (TME) in the three subtypes. **A**–**D** Immune infiltration comparison for the three subtypes using MCP-counter, TIMER, quanTIseq, and CIBERSORT, respectively. *P*-values were showed as: *P < 0.05: **P < 0.01; ***P < 0.001; ****P < 0.0001

The infiltration of different immune cells in the sample constitutes the TME. First, we used the MCP-counter, TIMER, quantTIseq, and CIBERSORT algorithms to score the degree of immune cell infiltration in each HCC sample. The abscissas of Fig. 6A–D show the immune cell types with immune infiltration differences between the three subtypes obtained by these four algorithms. All these algorithms revealed that the macrophage infiltration score of Cluster 3 was higher than that of the other two types. The quanTIseq and CIBERSORT algorithms demonstrated that Cluster 3 had higher infiltration scores of regulatory T (Treg) cells than the other two types (MCP-counter and TIMER algorithms could not evaluate the infiltration of Treg cells, Fig. 6C, D). Regarding B cells, the MCP-counter, TIMER, and quanTIseq algorithms considered Cluster 3 to score higher than Cluster 1 (Fig. 6A–C). Moreover, the MCP-counter, TIMER, and quanTIseq algorithms considered Cluster 1 neutrophil infiltration scores higher than Cluster 3 (Fig. 6A–C). Regarding CD8 + T cells, the primary mediator of anti-cancer immunity, only the quanTIseq algorithm showed a higher infiltration score in Cluster 3 (Fig. 6C), and there was no statistical significance in the other three algorithms.

### Expression of immune checkpoint inhibitor genes and N^6^-methyladenosine regulatory genes

**Fig. 7:**
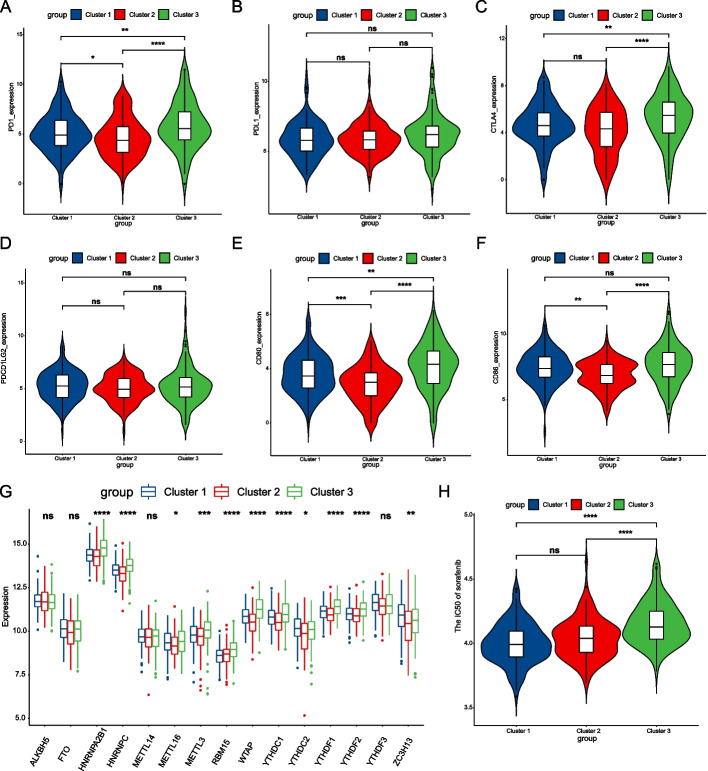
The expression of immune checkpoint inhibitor genes and m6A-regulated genes between the three subtypes. **A**–**F** The respective expression of PD1, PDL1, CTLA4, PDCD1LG2, CD80, and CD86 were compared in the three subtypes. **G** Comparison of expression levels of 15 m6A regulatory genes between the three subtypes. **H** Comparison of drug sensitivity of sorafenib between the three subtypes. *P*-values were showed as: *P < 0.05: **P < 0.01; ***P < 0.001; ****P < 0.0001; ns, no significance

As shown in Fig. 7B, D, the expression levels of *CD274* (*PDL1*) and *PDCD1LG2* (*PDL2*) among the three subtypes were not statistically significant. In comparing the expression levels of the remaining four genes, *PDCD1* (*PD1*), *CTLA4*, *CD80*, and *CD86*, we found that Cluster 3 was significantly higher than the other two types (Fig. 7A, C, E, F).

The analysis of expression levels of the m6A-regulated genes indicated that the expression levels of *ALKBH5*, *FTO*, *METTL14*, and *YTHDF3* were not statistically significant among the three subtypes; *YTHDC2* and *ZC3H13* were more expressed than in Cluster 1; the remaining nine genes, including *YTHDF2*, *YTHDF1*, *METTL16*, *METTL3*, *YTHDC1*, *WTAP*, *RBM15*, *HNRNPA2B1*, and *HNRNPC*, with high expression in Cluster 3 (Fig. 7G).

### Prediction of drug sensitivity and exploration of the molecular mechanism

By comparing the susceptibility of the three subtypes to sorafenib (Fig. 7H), we found no statistical significance between Cluster 1 and Cluster 2, and there were noteworthy differences between Cluster 1 and Cluster 2 (*P* < 0.0001) and between Cluster 1 and Cluster 3 (*P* < 0.0001). This suggests that Cluster 3 is more insensitive to Sorafenib than Clusters 1 and 2.

**Fig. 8:**
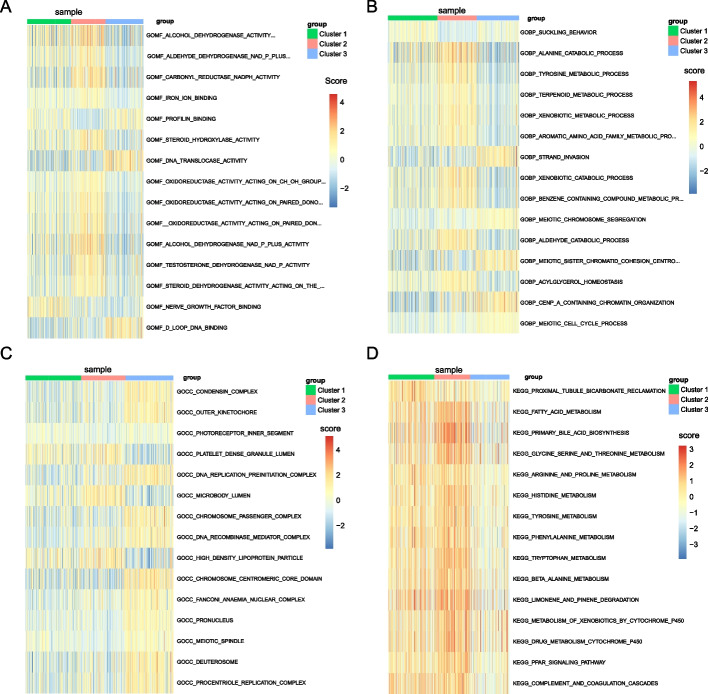
The analysis of Gene Ontology (GO) enrichment and Kyoto Encyclopedia (KEGG) pathway using the gene set variation analysis (GSVA) in the three subtypes. **A**–**C** Heatmaps for molecular function (MF), biological process (BP), and cellular component (CC) in GO enrichment analysis by the GSVA. **D** The heatmap from KEGG pathway analysis using GSVA

We next executed GO and KEGG analysis on these three subtypes using the method of GSVA. The differences between these three subtypes in molecular function (MF) were mainly in the activities of multiple reductases and dehydrogenases (Fig. 8A). The differences in the biological processes (BPs) of the three subtypes were mainly catabolic processes and meiosis-related processes of various substances (Fig. 8B). The differences in these three subtypes' cellular components (CC) were primarily centered on chromosome-related structures (Fig. 8C). Regarding the KEGG pathway, the discrepancies between the three subtypes mainly were the metabolic processes of multiple substances, especially the metabolism of various amino acids (Fig. 8D).

### Establishment of the cuproptosis-based prognostic signature score

Based on the analysis of TMB, TME, and the immune checkpoint inhibitor genes, we believe that Cluster 3 is more sensitive to immunotherapy, and immunotherapy is less effective for Cluster 1. Furthermore, the survival analysis showed a significant difference in overall survival between Cluster 1 and Cluster 3 (*P* = 0.016, Fig. 2D). Therefore, we tried to use the DEGs between the two subtypes to construct a prognostic scoring model and employ this model to predict the efficacy of immunotherapy.

**Fig. 9:**
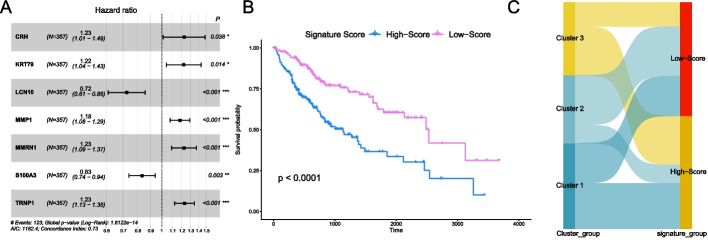
The identification of the signature score. **A** The hazard ratio plot for seven genes (Greater than 1 is the risk factor, less than 1 is the protective factor, and the farther away from 1, the stronger the degree). **B** Survival curves of high signature score group and low signature score group (grouped according to the median). **C** The Sankey diagram showing the relationship between the cluster grouping and the signature score grouping

The DEGs distribution *of Cluster 3 respect to Cluster 1* is shown in Additional file 2: Fig. S2C. We next screened these DEGs using LASSO regression (Additional file 2: Fig. S2D, E), yielding 23 genes that were subsequently used for univariate Cox (*P* < 0.2) and multivariate cox regression (*P* < 0.05). Finally, we determined a prognostic scoring model based on seven genes, among which *LCN10* and *S100A3* were protective, and *CRH*, *KRT79*, *MMP1*, *MMRN1*, and *TRNP1* were risk genes (Fig. 9A). The formula for the prognostic score is as follows:

$$\begin{aligned} {\text{Signature}}\,{\text{Score}}\, = \, & {1}.{2273749} \times {\text{CRH}}\, + \,{1}.{2221668} \times {\text{KRT79}}\, + \,0.{7244353} \times {\text{LCN1}}0 \\ & + \,{1}.{18217}0{7} \times {\text{MMP1}}\, + \,{1}.{2262966} \times {\text{MMRN1}}\, + \,0.{8335867} \times {\text{S1}}00{\text{A3}} \\ & + \,{1}.{2312168} \times {\text{TRNP1}}. \\ \end{aligned}$$

**Fig. 10:**
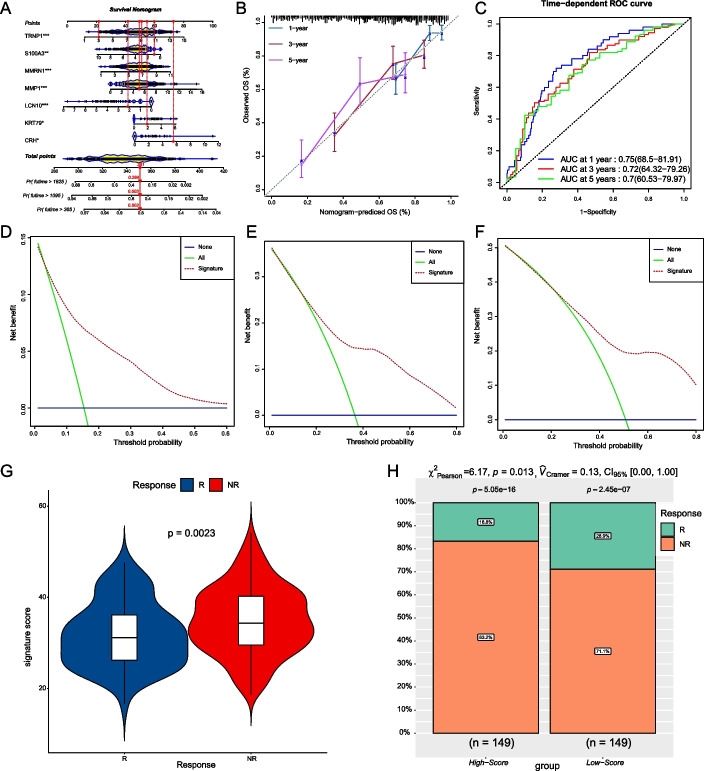
Validation of the signature score model. **A** The Nomogram to predict the signature score model's 1-, 3-, and 5-years survival. **B**, **C** Calibration curves and time-dependent ROC curves for the signature score model's 1-, 3-, and 5-year survival. **D**–**F** 1-, 3-, and 5-year decision curve analysis (DCA) plots of the signature score model. **G** Comparison of the signature score of immunotherapy responders (R) and non-responders (NR) in the IMvigor210 cohort. **H** The comparison of the immune efficacy of the two groups in the IMvigor210 cohort (grouped according to the median of the signature score)

HCC patients were divided into high-score and low-score groups according to the median of their signature scores, and the overall survival of these two groups was statistically significant (*P* < 0.0001, Fig. 9B). We next drew a Sankey diagram to show the relationship between the results grouped by consensus clustering and the results grouped by signature score (Fig. 9C). Figure 10A shows the nomogram of this scoring model we plotted using the “regplot” R package. This nomogram can be used to predict the probability of survival of patients with HCC greater than 1 year, 3 years, and 5 years. Calibration curves plotted by the “caret” R package show the use of this scoring model to predict the deviation of HCC patients’ 1-, 3-, and 5-years survival status from the actual status (Fig. 10B). Time-dependent ROC curves and their AUC plotted by the “timeROC” R demonstrate the accuracy of using this scoring model to predict 1-, 3-, and 5-years survival status of HCC patients (Fig. 10C). Figure 10B, C show that the scoring model has satisfactory accuracy. The DCA was plotted by the “rmda” R package, which is widely used to evaluate the performance of models in supporting decision-making. NONE and ALL are two reference lines, and the closer the curve of the model is to the two reference lines, the less useful it is. The DCA of 1 year, 3 years, and 5 years showed that the scoring model has adequate clinical utility (Fig. 10D–F). Next, we use the signature score model to score the IMvigor210 cohort. Figure 10G reveals that the signature score between the immunotherapy responders (R) and the immunotherapy non-responders (NR) was statistically significant (*P* = 0.0023). The IMvigor210 cohorts were divided into two groups based on the median of the signature score, and the high-score group was more sensitive to immunotherapy than the low-score group. (*P* = 0.013, Fig. 10H).

## Discussion

Due to late diagnosis, chemotherapy resistance, frequent recurrence, and metastasis, HCC is still one of the most malignant cancers. With the continuous research on immunotherapy and targeted therapy for HCC, the prognosis of HCC has been significantly improved [31]. Therefore, exploring multiple methods to assess the sensitivity of patients to immunotherapy is of great significance for the treatment of HCC. At the same time, the rapid development of science and technology and the wide application of systems biology technologies such as genomics and proteomics have provided new methods and ideas for the classification, individualized treatment, curative effect monitoring, and prognosis judgment for HCC. There are few studies between cuproptosis and HCC, so the classification of HCC based on CRGs and the exploration of molecular characteristics among these subtypes will create new analytical ideas for HCC.

This study used a consistent clustering approach to classify 357 HCC patients into three subtypes based on ten CRGs. Survival analysis, t-SNE, and UMAP indicated that the classification results were clinically meaningful and had good discrimination. We constructed predictive models using six supervised machine learning approaches to identify these three subtypes in the clinic and ranked the importance of genes affecting the identification of Cluster 3. Next, we explored the differences between these three subtypes in gene mutation, TMB, TME, expression of immune checkpoint inhibitor genes and N6-methyladenosine regulatory genes, and molecular mechanisms. We thought that the sensitivity to immunotherapy differed significantly between Cluster 1 and Cluster 3, so we constructed a prognostic score model based on the differential genes of these two subtypes. This scoring model was statistically significant in evaluating the efficacy of immunotherapy in the IMvigor210 cohort.

In the CatBoost model identifying Cluster 3, *CDKN2A*, *GLS*, and *FDX1* were significantly more important than the remaining seven genes. *CDKN2A* and *GLS* are repressor genes during cuproptosis. *CDKN2A* was previously thought to induce cell cycle arrest in both G1 and G2 phases, and its inactivation is an important event in many cancer types [32–34]. *GLS* was thought to catalyze the first reaction in the main pathway of renal catabolism of glutamine and regulate levels of the neurotransmitter glutamate [35]. *FDX1*, a critical upstream promoter in cuproptosis [8], was shown in previous studies to be essential for synthesizing various steroid hormones and involved in mitochondrial cytochrome P450, which reduces steroidogenesis [36, 37]. According to our gene expression heat map (Fig. 3A), *GLS* has the highest expression in Cluster 3 and the lowest in Cluster 2; *CDKN2A* has the highest expression in Cluster 3 and the lowest in normal liver tissue; *FDX1* has the lowest expression in Cluster 3 and the highest expression in Cluster 2. Combined with the expression of the remaining seven genes, we believe that the activity of cuproptosis in cluster 3 is lower than that in clusters 1 and 2, and cuproptosis in normal liver tissue is active. Moreover, according to the univariate survival analysis of these ten CRGs, high expression of all cuproptosis suppressor genes (*MTF1*, *GLS*, *CDKN2A*) results in decreased overall survival. Consequently, the decrease in cuproptosis activity may be one of the crucial reasons for the poor prognosis of Cluster 3.

The gene mutation analysis found that the proportion of *TP53* mutation in Cluster 3 was significantly higher than that in the other two subtypes. *TP53* acts as a tumor suppressor in many tumor types and induces growth arrest or apoptosis depending on the physiological circumstances and cell type [38, 39]. Mutational inactivation of *TP53* may be associated with reduced cuproptosis activity. This speculation requires further experimental exploration. According to the results of the TMB comparison, Cluster 2 and Cluster 3 may be more sensitive to immunotherapy than Cluster 1, and there may be no difference in the sensitivity of Cluster 2 and Cluster 3 to immunotherapy.

Regarding the relationship between cuproptosis and immunotherapy, several studies have demonstrated that Treg cells and macrophages are the primary cells responsible for immune dysfunction in HCC [40, 41]. The tumor microenvironment analysis found that Cluster 3 had significantly higher Treg cells and macrophages scores, suggesting a possible immunosuppressive effect in Cluster 3. In the analysis of immune checkpoint inhibitor genes, we found that *PDCD1 (PD1)*, *CTLA4*, *CD80*, and *CD86* were more expressed in Cluster 3 than the other two subtypes. This result may suggest that Cluster 3 is more sensitive to immunotherapy than Clusters 1 and 2. In addition, the TMB of Cluster 3 is higher. These analyses imply that immunotherapy may achieve better therapeutic effects in Cluster 3, the subtype with low cuproptosis activity. Therefore, the link between cuproptosis activity and immunotherapy sensitivity is an aspect worthy of further exploration.

In the drug sensitivity analysis, Cluster 3 was not as sensitive to sorafenib as Clusters 1 and 2, suggesting that combined immunotherapy and small-molecule receptor tyrosine kinase inhibitors are less effective than expected for this subtype.

However, there are some limitations to our study. The conclusions of this study are based on bioinformatics and machine learning analysis of public databases, lacking support from basic experiments and clinical cohorts. Besides, there are few relevant studies on the relationship between cuproptosis and HCC, which leads us to some inferences that may not be accurate enough. We built machine learning models to identify different subtypes, but these models were not made into a convenient and fast web tool and only ran on R.

## Conclusions

Overall, we used a machine learning approach to classify HCC into three subtypes based on the expression of CRGs and explored the different molecular signatures of the three subtypes. These explorations may provide new ideas and insights for the treatment and research of HCC. Finally, we constructed a scoring model that can predict the overall survival of HCC, and the score can be used to predict the efficacy of immunotherapy in the IMvigor210 cohort. This scoring model may play a role in studying the relationship between immunotherapy and cuproptosis.

## Supplementary Information

**Additional file1**. **Fig S1**: The flow chart of this study. HCC, hepatocellular carcinoma. TMB, tumor mutation burden. TME, tumor microenvironment. GSVA, gene set variation analysis. GO, Gene Ontology. KEGG, Kyoto Encyclopedia.

**Additional file2**. **Fig S2**: Association analysis of ten cuproptosis-related genes (CRGs), and DEGs screening of Cluster 3 and Cluster 1. (A) Protein interaction relationships of the ten CRGs (the larger the area of the circle, the stronger the association with other genes). (B) The correlations plot of the ten CRGs. (C) The volcano map of differentially expressed genes (DEGs) between Cluster 1 and Cluster 2. (D) Changes in the trajectory of each independent variable of LASSO regression. (F) log value of the independent variable lambda of LASSO regression.

**Additional file3**. **Table S1**: Six immune checkpoint inhibitor genes and 15 m6A regulatory genes.

**Additional file4**. **Table S2**: Clinical features of the three subtypes derived from consensus clustering.

## Data Availability

Data from the public databases were used in this study. Data can be found here: http://xena.ucsc.edu/ and http://research-pub.gene.com/IMvigor210CoreBiologies/. If you need the CatBoost prediction model mentioned in the article, please get in touch with the corresponding author.

## Abbreviations

HCC: Hepatocellular carcinoma
CRGs: Cuproptosis-related genes
DEGs: Differentially expressed genes
PPIs: Protein–protein interactions
TMB: Tumor mutation burden
TME: Tumor microenvironment
IC50: 50% Inhibiting concentration
LR: Logistic regression
RF: Random forest
SVM: Support vector machine
NNET: Neural network
GSVA: Gene set variation analysis
GO: Gene ontology
KEGG: Kyoto Encyclopedia

## Footnotes

1 http://xena.ucsc.edu/.

2 https://www.genecards.org/.

3 http://www.gsea-msigdb.org/gsea.
