# Supplementary material for: Identification of the cuproptosis-related molecular subtypes and an immunotherapy prognostic model in hepatocellular carcinoma
Source: BMC Bioinformatics. 2022 Nov 16;23:485. doi: 10.1186/s12859-022-04997-0 (PMC9667659; doi:10.1186/s12859-022-04997-0)
Supplement: Supplementary file 1 — Additional file1. Fig S1: The flow chart of this study. HCC, hepatocellular carcinoma. TMB, tumor mutation burden. TME, tumor microenvironment. GSVA, gene set variation analysis. GO, Gene Ontology. KEGG, Kyoto Encyclopedia. [file 12859_2022_4997_MOESM1_ESM.pdf]

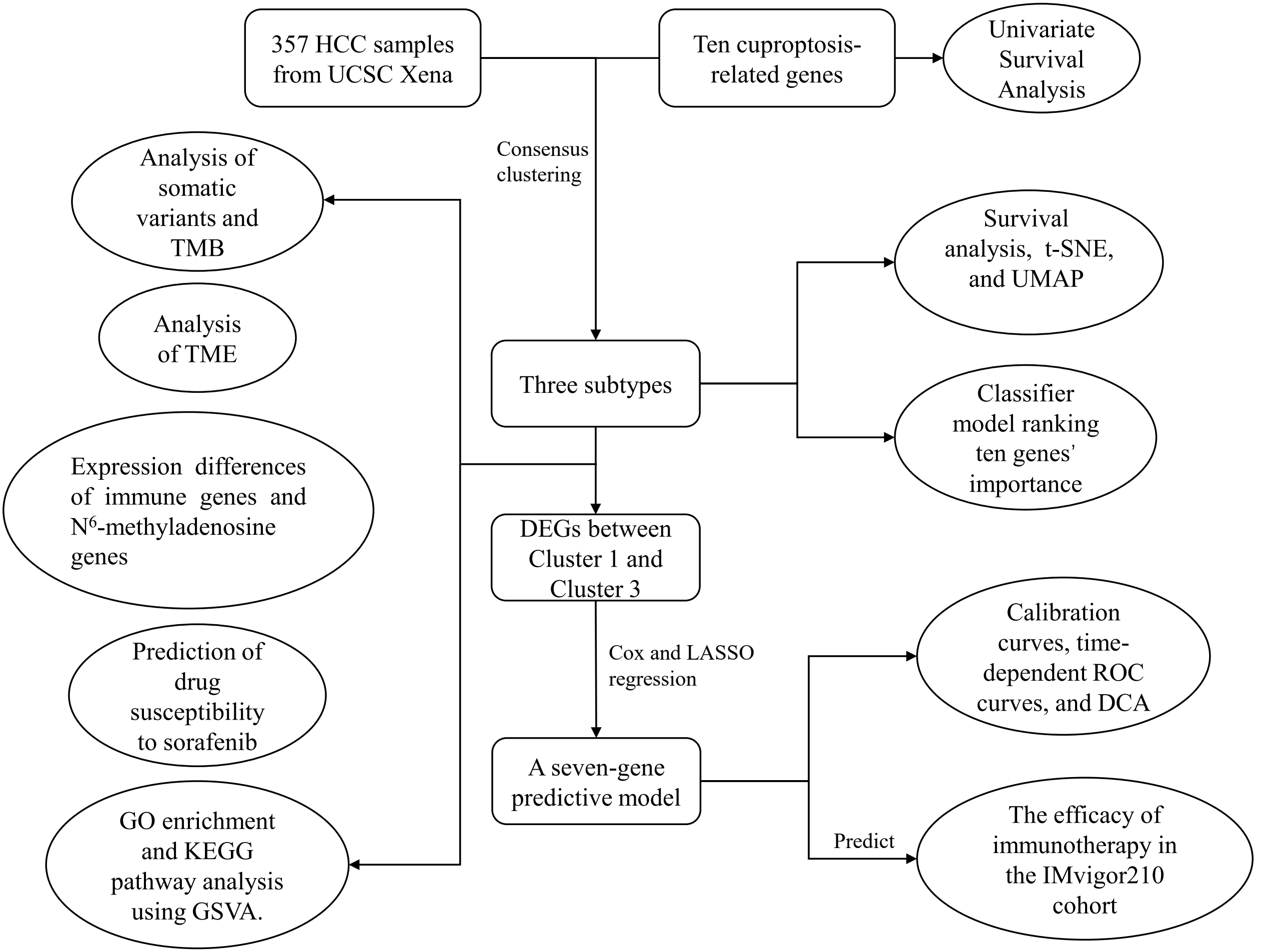

Supplementary Fig. 1 The flow chart of this study. HCC, hepatocellular carcinoma. TMB, tumor mutation burden. TME, tumor microenvironment. GSVA, gene set variation analysis. GO, Gene Ontology. KEGG, Kyoto Encyclopedia.
