# Supplementary material for: Identification of the cuproptosis-related molecular subtypes and an immunotherapy prognostic model in hepatocellular carcinoma
Source: BMC Bioinformatics. 2022 Nov 16;23:485. doi: 10.1186/s12859-022-04997-0 (PMC9667659; doi:10.1186/s12859-022-04997-0)
Supplement: Supplementary file 2 — Additional file2. Fig S2: Association analysis of ten cuproptosis-related genes (CRGs), and DEGs screening of Cluster 3 and Cluster 1. (A) Protein interaction relationships of the ten CRGs (the larger the area of the circle, the stronger the association with other genes). (B) The correlations plot of the ten CRGs. (C) The volcano map of differentially expressed genes (DEGs) between Cluster 1 and Cluster 2. (D) Changes in the trajectory of each independent variable of LASSO regression. (F) log value of the independent variable lambda of LASSO regression. [file 12859_2022_4997_MOESM2_ESM.pdf]

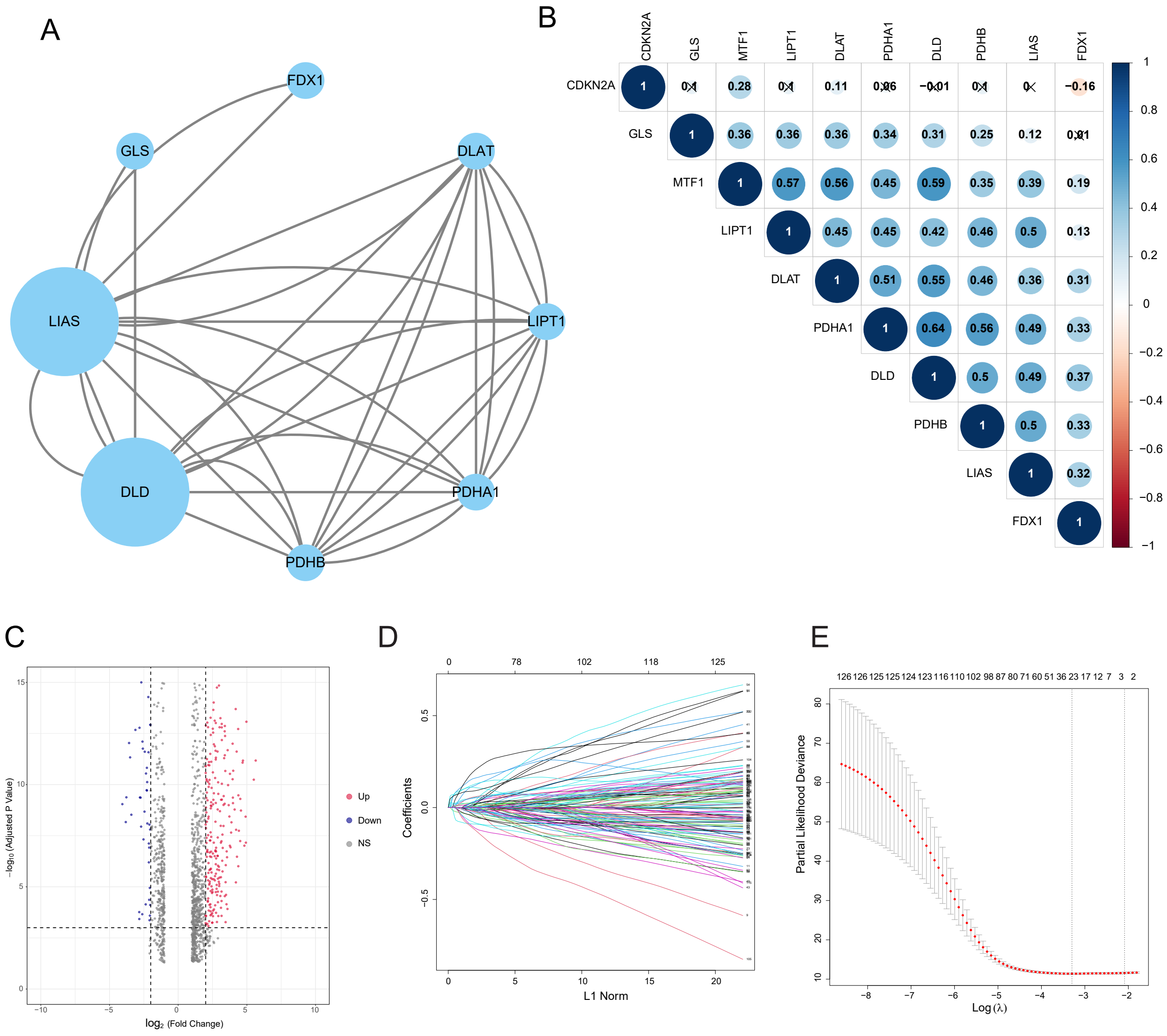

Supplementary Fig. 2 Association analysis of ten cuproptosis-related genes (CRGs), and DEGs screening of Cluster 3 and Cluster 1. **(A)** Protein interaction relationships of the ten CRGs (the larger the area of the circle, the stronger the association with other genes). **(B)** The correlations plot of the ten CRGs. **(C)** The volcano map of differentially expressed genes (DEGs) between Cluster 1 and Cluster 2. **(D)** Changes in the trajectory of each independent variable of LASSO regression. **(F)** log value of the independent variable lambda of LASSO regression.
