# Supplementary material for: Identification of the cuproptosis-related molecular subtypes and an immunotherapy prognostic model in hepatocellular carcinoma
Source: BMC Bioinformatics. 2022 Nov 16;23:485. doi: 10.1186/s12859-022-04997-0 (PMC9667659; doi:10.1186/s12859-022-04997-0)
Supplement: Supplementary file 3 — Additional file3. Table S1: Six immune checkpoint inhibitor genes and 15 m6A regulatory genes. [file 12859_2022_4997_MOESM3_ESM.pdf]

Six immune checkpoint inhibitor genes

PDCD1
CD274
PDCD1LG2
CTLA4
CD80
CD86

Fifteen m6A regulatory genes

YTHDF2
YTHDF3
YTHDF1
METTL16
METTL3
YTHDC2
YTHDC1
METTL14
WTAP
RBM15
FTO
HNRNPA2B1
HNRNPC
Z13H13
ALKBH5
Z13H13
