# Supplementary material for: Identification of the cuproptosis-related molecular subtypes and an immunotherapy prognostic model in hepatocellular carcinoma
Source: BMC Bioinformatics. 2022 Nov 16;23:485. doi: 10.1186/s12859-022-04997-0 (PMC9667659; doi:10.1186/s12859-022-04997-0)
Supplement: Supplementary file 4 — Additional file4. Table S2: Clinical features of the three subtypes derived from consensus clustering. [file 12859_2022_4997_MOESM4_ESM.pdf]

Comparison of clinical characteristics of patients in the three subtypes

Characteristic	Cluster 1, N = 135[1]	Cluster 2, N = 107[1]	Cluster 3, N = 115[1]	p-value[2]
Age				<0.001
Mean (SD)	56.4 (15.8)	63.7 (9.9)	58.8 (11.8)	
Median (IQR)	58.0 (46.0, 68.0)	65.0 (57.0, 70.5)	60.0 (51.0, 67.0)	
T				NA
T1	78 (58%)	59 (55%)	37 (32%)	
T2	25 (19%)	27 (25%)	37 (32%)	
T3	28 (21%)	18 (17%)	32 (28%)	
T4	2 (1.5%)	2 (1.9%)	9 (7.8%)	
TX	2 (1.5%)	1 (0.9%)	0 (0%)	
N				0.519
N0	88 (65%)	71 (66%)	82 (71%)	
N1	3 (2.2%)	0 (0%)	1 (0.9%)	
NX	44 (33%)	36 (34%)	32 (28%)	
M				0.327
M0	92 (68%)	75 (70%)	89 (77%)	
M1	2 (1.5%)	0 (0%)	1 (0.9%)	
MX	41 (30%)	32 (30%)	25 (22%)	
Stage				NA
stage I	72 (53%)	56 (52%)	36 (31%)	
stage II	22 (16%)	26 (24%)	34 (30%)	
stage III	29 (21%)	17 (16%)	37 (32%)	
stage IV	3 (2.2%)	0 (0%)	1 (0.9%)	
stage X	9 (6.7%)	8 (7.5%)	7 (6.1%)	
Gender				0.002
female	55 (41%)	21 (20%)	41 (36%)	
male	80 (59%)	86 (80%)	74 (64%)	

[1] n (%)

[2] Kruskal-Wallis rank sum test; Fisher's exact test; Pearson's Chi-squared test
